# 743 Changes in Epidemiology and Outcomes in a National Burn Centre over 17 Years (2006–2023)

**DOI:** 10.1093/jbcr/irae036.286

**Published:** 2024-04-17

**Authors:** Anna Dargan, Richard B Wong She, Paul Baker, Lindsay Damkat-Thomas

**Affiliations:** Middlemore National Burns Centre, Middlemore Hospital, Auckland, Auckland; National Burns Centre, Middlemore Hospital, Auckland, Auckland; Middlemore National Burns Centre, Middlemore Hospital, Auckland, Auckland; National Burns Centre, Middlemore Hospital, Auckland, Auckland; Middlemore National Burns Centre, Middlemore Hospital, Auckland, Auckland; National Burns Centre, Middlemore Hospital, Auckland, Auckland; Middlemore National Burns Centre, Middlemore Hospital, Auckland, Auckland; National Burns Centre, Middlemore Hospital, Auckland, Auckland

## Abstract

**Introduction:**

We reviewed the trends in referrals, admissions, treatment and outcomes for the burn patients managed in a National Burn Center (NBC) over a 17-year period since the center opened in 2006. Nationwide, there are four burn centres which care for patients who fulfil the American Burn Association criteria for a referral to a burn center. The NBC is located in the largest metropolitan area, and takes additional referrals of “severe” burn injuries (e.g. >30% total burn surface area (TBSA)). We will explore the burden of referrals and present the evolving models of care over the time period. Our total catchment population of 5.1 million people spread over 268, 021 km2 compares to the average population of a US state of 5.7 million over 182, 949 km2.

**Methods:**

Patient data was extracted from the departmental database which has been prospectively recorded since the center opened in 2006. All inpatients received multidisciplinary team care.

**Results:**

Between 2006-2023 there were 6279 admissions, of which 307 were burns >30% TBSA. We observed an over-representation of some ethnic groups with burn injury relative to the population, specifically Māori and Pasifika.

Reviewing the admissions trends, there was a 12% increase in admissions comparing the three-year period of 2006-2009 to 2020-2023. Admissions increased by 21% in patients with < 10% TBSA from an average of 289 patient per year in 2006-2009, to 348 patients per year in 2020-2023. Admissions for patients with >10% TBSA decreased from an average of 59 per year in 2006-2009 to 43 per year in 2020-2023. Despite the overall increase in admission numbers, the average percentage of total admissions that were from national/out-of-region decreased slightly from 6.2% in 2006-2009 compared to 4.7% in 2020-2023. This is a result of being outweighed by a much larger contribution from the local catchment area.

The average length of stay for patients increased slightly from 7.29 days (2006-09) to 7.76 days (2020-2023). There was a 19% increase in average operations from 1378 to 1633 year, and a 34% increase in average operating minutes per year (2006-09 vs 2020-23), indicating an increased operative burden on the unit.

**Conclusions:**

Centralisation of care is reported to improve the standard of care in complex burns. From 2006-2023, our team has observed increasing numbers of patients accepted that do not meet the threshold for the agreed National Burn referral criteria, suggesting increasing reliance on referring services. Our findings have also shown that increased demand for a service can result from the demand generated from the local population independent of referring services, therefore the location of a NBC should take into account not only geographical distance and accessibility, but also local population changes.

**Applicability of Research to Practice:**

This paper reflects the changing practice in burns management following the establishment of a National Burn Center/centralised burn care model, and the evolving models of care in burns.

